# ‘I don’t want him to always be so far behind’: Parental perceptions of child independence in the context of extreme prematurity; a qualitative study

**DOI:** 10.1177/13674935241256545

**Published:** 2024-05-27

**Authors:** Emmi Suonpera, Katie Gallagher, Neil Marlow, Anne Lanceley

**Affiliations:** UCL EGA Institute for Women’s Health, 4919 University College London, London, UK

**Keywords:** Adolescent, developmental disabilities, extremely preterm infants, parenting, qualitative research

## Abstract

This study addresses the paucity of research on parents of extremely preterm adolescents (born <27 weeks of gestation) and their experiences within the framework of parental determinism. We conducted semi-structured interviews with twenty-two mothers and one father. Data were analysed thematically, revealing three overarching themes and eight subthemes shaping parental accounts. These themes centred on parental ambitions for their children, their perceptions of their child’s abilities, and the parenting behaviours employed to support parental aspirations. Parents’ actions were influenced by their ambitions and the belief that they could impact their child’s future independence. While some parents adopted ‘trusting’, non-intensive parenting behaviours, those anticipating challenges for their child’s future independence resorted to intensive parenting practices. These findings align with the concept of parental determinism, emphasising the perceived causal link between present parental actions and future child outcomes. In the context of extreme prematurity, a nuanced understanding of parental perceptions regarding their child’s future independence aligned with a delicate balance between hope and realistic aspiration is crucial for enhancing parental support and well-being.

## Introduction

Extremely preterm (EP) infants face extended hospitalisation in neonatal intensive care units (NICUs), leading to parental concerns about their baby’s survival and future health and development (Baia et al., 2016; Caporali et al., 2020). Such concerns are warranted; at 12 years of age 38% of survivors born at <27 weeks of gestation during 2006 in England had moderate to severe neurodevelopmental disabilities relating to cognition, manual abilities, motor skills, vision, and hearing (Marlow et al., 2021). While many EP survivors enjoy good outcomes and quality of life, preterm birth is associated with persistent cognitive impairments (O'Reilly et al., 2020) and emotional and behavioural challenges (Taylor, 2020), impacting EP adolescents transition to autonomous adulthood and fostering prolonged family dependence (Hallin et al., 2010).

Following hospital discharge, parents bear primary responsibility for their EP child’s long-term outcomes, although intermittent medical follow-up care extends to age two or even through school-age in some settings (National Institute for Health and Care Excellence, 2018). Parents of younger preterm children have expressed difficulties in ‘trusting’ their child’s health due to their preterm birth (Schuetz Haemmerli et al., 2020) leading to long-term distress, especially among mothers (Yaari et al., 2019). The association of prematurity with various physical, psychological, and cognitive health concerns can prolong uncertainty in child development. When approaching school-age, parents of EP children may feel less confident of their child’s capabilities compared to parents of full-term children (Taylor, 2020). For EP children with severe disabilities, the resulting parental role is characterised by extensive caregiving efforts because of the child’s complex care needs (Wilson and Cook, 2018).

By early adolescence, new expectations such as increased emotional regulation and social competence, appear as autonomous future adult functioning with independence from the family becomes topical, and the importance of adolescent peer-relationships is heightened (Casey, 2015). Simultaneously, symptomology of potential other morbidities such as educational issues and anxiety may become clearer (Hallberg et al., 2009). In this context, EP families may experience unanticipated new challenges while parents become increasingly aware of their child’s future independence (Moore et al., 2006).

Modern Western parenting culture emphasises parental responsibility in child socialisation (Nomaguchi and Milkie, 2020). Parenting is seen as a goal-oriented effort to cultivate valuable adult skills to raise a ‘successful’ adult (Faircloth, 2020). Cultural models of adult competence, defining the skills needed to thrive as an independent adult (i.e. to ‘make it’ in the society and/or ‘function’ independently), guide parenting behaviour (Ogbu, 1981: 410). Parents aim to ensure their child’s optimal development in alignment with the culturally valued adult competence. Consequently, parental ambitions shape parenting practices, influencing child development (Aytac et al., 2016; Keller et al., 2016). For instance, in cultures valuing adult independence, parents prioritise socialisation strategies that foster early independent functioning (Keller et al., 2016).

A key aspect of this discursive understanding of ‘parenting’ is parental determinism; the concept that parental behaviour has direct causal impact on a child’s future success (Füredi, 2008: 55-61). Parenting behaviour is portrayed as the most critical factor in ensuring a child’s future competence as an adult (Faircloth, 2020). For example, healthcare professionals working with children increasingly highlight parenting behaviour’s significance as a salient determinant of child outcomes (Yerkes et al., 2019). Consequently, parental determinism can be regarded to influence the wider social context in which contemporary Western parents act (Dermott, 2012; Füredi, 2008: 55-61). This belief in optimising child outcomes as primarily the parents’ responsibility encourages intensive parenting by suggesting that intensive parental involvement is ‘essential’ for maximising child outcomes (Yerkes et al., 2019) and that early and substantial investments in a child’s future yield greater returns (Gillies, 2020).

Although quantitative long-term EP family outcomes have been reported (Suonpera et al., 2023), there is a lack of research on the experiences of parents of preterm adolescents, especially among those born at the earliest gestational ages (Wilson and Cook, 2018) warranting a qualitative investigation of parental experiences. This article explores how parents construct ‘parenting’ their EP born child entering adolescence and how parents perceive their parental roles and view their parenting behaviours.

## Aim

The aim was to uncover the influence of the prevailing societal model of parental determinism on the experiences of parents of EP children reaching adolescence and to eventually improve parental support leading to better well-being for these parents.

## Methods

This study used interpretive qualitative methodology with semi-structured interviews to understand how parents describe their experiences of caring for their EP child entering adolescence. Theoretically, it focused on the social construction of ‘parenting’ in participants’ accounts of their roles, actions, and decisions (Marvasti, 2019). The interpretive approach best fitted the study by uncovering both individual experiences and shared contextual meaning patterns in parental accounts (Guba and Lincoln, 1994).

This inquiry is part of a birth cohort study in England focussing on extreme prematurity and long-term health since 2006 (Marlow et al., 2021). Families with infants born <27 weeks of gestation were recruited at birth from maternal units across England and underwent assessments at 2.5 and 12 years of age. This study represents the first qualitative exploration of parental experiences within the cohort study.

The interviewer, a clinical assessor in the cohort study, had prior knowledge of the interviewees’ family backgrounds. Positionally, she was a younger, non-parent female researcher without personal experience of prematurity. The study follows the consolidated criteria for reporting qualitative research (Tong et al., 2007).

### Sampling and recruitment

Parents were invited to participate in the study interview if they consented to their child’s clinical assessment. Recruitment involved purposeful case selection to ensure maximum variation based on child’s sex, gestational age, disability level (established by the clinical child assessment), and family demographics. *A priori,* a sample size of 20–24 was considered sufficient to achieve data saturation when no new perspectives would be introduced. All contacted parents agreed to participate. Ethical approval was granted by the UCL Research Ethics Committee (reference: 10175/001). Each parent gave written informed consent, which was verbally confirmed during the interview.

### Interview

The interview schedule followed a pre-designed topic-guide themed around the context of family life, child characteristics, caring experiences, and parents’ experiences of the child’s transition to secondary school (Table 1). The topic-guide development and finalisation were supported by literature review (e.g. Hallberg et al., 2009; Hetherington et al., 2010; Kantrowitz-Gordon et al., 2016; Scorgie et al., 2004), by the study advisory group formed of parents and charity representatives, and by piloting with three parents of full-term born children of similar ages. The interview schedule did not include explicit questions about the EP birth. Instead of setting the birth as a starting point for the interview, the topic-guide gave an opportunity for ‘prematurity’ to emerge spontaneously from the parents’ accounts. All interviewees were identified with a number and were given pseudonyms. The interviews were carried out in English language, audio recorded, and transcribed verbatim. Later, parents’ quotes were edited without changing meaning to improve clarity. Field notes were kept. Transcripts were returned to the participants for comments and/or corrections, if wished. The participants did not provide feedback on the findings of this study.

**Table 1.** Interview topic guide with example questions for interviews with parents of extremely preterm born early adolescents.

Topic	Example questions
Family context	Could you tell me about your family/‘make up’ of your family/family’s daily life?
Child characteristics	If you think about (child’s name) how would you describe her/him as a child?
	If you were to think about (child’s name) as a child, is there something that has impacted your parenting?
Transition to secondary school/adolescence	(Child’s name) has started secondary school/will start soon, is that something you think about?
	Do you have any particular ambitions for your child?
	Are there any challenges in reaching those ambitions?
	How do you support (child’s name) in reaching those goals?
Parental experiences	Are there things that particularly influence your parenting?
	Do you have priorities as a parent? – Something that has always been important for you in your role as a parent?
	Is there something that you find challenging in your parenting?
	What would you need to achieve these goals/priorities?
Parental support	What do you feel you need as a parent to assist you through the transition years?
	Do you receive support in your role as a parent?
	Do you share responsibilities with your partner?
	Do you see that you and your partner have similar roles in parenting?

### Data analysis

A step-wise thematic analysis of the interview corpus to identify themes as patterns within the data to analyse and report them as outlined by Braun and Clarke (2006) was conducted by the first author using NVivo Pro 12 (Lumivero) supported by repeated review and discussions with the co-authors to refine the emerging themes. The following question was asked from the data: *How do parents describe experiences of parenting their child born EP during the transition to adolescence at around 12 years of age?*

### Trustworthiness

Study trustworthiness was supported by the steering committee guiding study design and conduct, interview topic guide piloting, and co-authors’ repeated review of the analysis results. Deviant cases are presented, and findings are reflected against relevant literature.

## Findings

### Demographics

Twenty-two semi-structured telephone interviews were conducted by ES between October 2017 and May 2018 at a time and date chosen by the parent, either from a private location at work or at home. In the interviews, lasting 14–65 minutes (excluding study introduction and closing remarks), some parents discussed matters freely following the first question relating to their family context covering questions in the subsequent topics, while in other parents’ interviews questions were asked in succession. A majority of the interviewees were women in their mid-forties who were married or living with a partner. Fourteen women were from white ethnic backgrounds, while two women’s ethnic backgrounds were mixed and six were from other, mainly Asian and African, ethnic backgrounds. The children of 12 mothers had a severe or moderate neurodevelopmental disability of whom three attended Special Educational Needs and Disabilities (SEND) schools or units. Many (16/22) women either worked part-time in paid employment or were full-time carers or homemakers while their partners were employed in intermediate or high-level professions (Table 2).

**Table 2.** Sociodemographic information for interviewed parents (*n* = 23).

Interview ID	Pseudonym	Age	Relationship status	Highest academic qualification	Working status	Highest SES in the household^a^	Gestational age in weeks	Child disability level^b^	School type	Siblings
1	Ms Beryl	≥40	Married	Post-secondary	Carer/homemaker	Unemployed	≥24	Severe	SEND	None
2	Ms Turquoise	≥40	Married	University degree	Carer/homemaker	High	≥24	Severe	SEND	1
3	Ms Chalcedony	≥40	Married	Secondary education	Part-time	High	≥24	None/mild	Secondary	None
5	Ms Sillimanite	≥40	Married	Post-secondary	Part-time	High	≥24	None/mild	Secondary	2
6	Ms Iolite	≥40	Married	Secondary education	Part-time	High	≥24	Severe	Junior	1
7	Ms Kyanite	≥40	Single	University degree	Carer/homemaker	Unemployed	≥24	None/mild	Junior	1
9	Ms Garnet	≥40	Married	University degree	Carer/homemaker	Intermediate	≥24	Moderate	Junior	1
10	Ms Quartz	≥50	Living with a partner	Secondary education	Part-time	Low	≥24	None/mild; moderate	Junior	1
11	Ms Fire Agate	≥40	Single	University degree	Part-time	Low	<24	Severe	Junior	2
13	Ms Ammolite	≥40	Married	University degree	Part-time	Intermediate	≥24	Severe	SEND	2
16	Ms Amethyst		Married		Full-time		≥24	None/mild	Junior	2
17	Ms Amazonite	≥40	Separated/divorced	Post-secondary	Full-time	High	≥24	None/mild	Secondary	None
18	Ms Onyx	≥40	Single	Secondary education	Carer/homemaker	Unemployed	≥24	Moderate	Junior	1
19	Ms Agate	≥40	Separated/divorced	University degree	Part-time	Intermediate	≥24	Moderate	Secondary	None
20	Ms Spinel	≥40	Separated/divorced	Secondary education	Full-time	Intermediate	≥24	None/mild	SEND	1
21	Ms Dioptase	≥30	Married	University degree	Part-time	High	≥24	None/mild	Junior	1
23	Ms Sphalerite	≥50	Living with a partner	University degree	Full-time	High	≥24	None/mild	Secondary	None
24	Ms Rhyolite	≥40	Single	University degree	Full-time	Self-employed	<24	Moderate	Secondary	1
25	Ms Jasper	≥50	Living with a partner	Post-secondary	Full-time	High	≥24	None/mild	Secondary	None
27	Ms Druzy	≥30	Married		Part-time	Low	≥24	None/mild	Secondary	3
28	Ms Aquamarine	≥30	Married	Post-secondary	Carer/homemaker	Low	≥24	Severe	Junior	1
29	Ms and Mr Jade	≥50	Married	University degree	Carer/homemaker	High	≥24	Moderate	Junior	1

^a^Derived using the United Kingdom National Statistics Socio-economic Classification (NS-SEC) simplified method (Office for National Statistics, 2016).

^b^Defined as (1) severe impairment: any one or more of an IQ score of more than −3 SD of full-term born comparator children, cerebral palsy with the Gross Motor Function Classification System (GMFCS) or the Manual Abilities Classification System (MACS) levels 3–5, and blindness or profound deafness; (2) moderate impairment: any one or more of an IQ score of < −2 SD to −3 SD of controls, GMFCS or MACS level 2, and visual impairment but not registered blind or wearing hearing aids but not with profound hearing loss; (3) children with less severe impairments or no functional limitations were categorised without or with mild impairment.

SD = standard deviation.

SEND = special educational needs and disabilities.

### Overall framework

**Figure 1.**
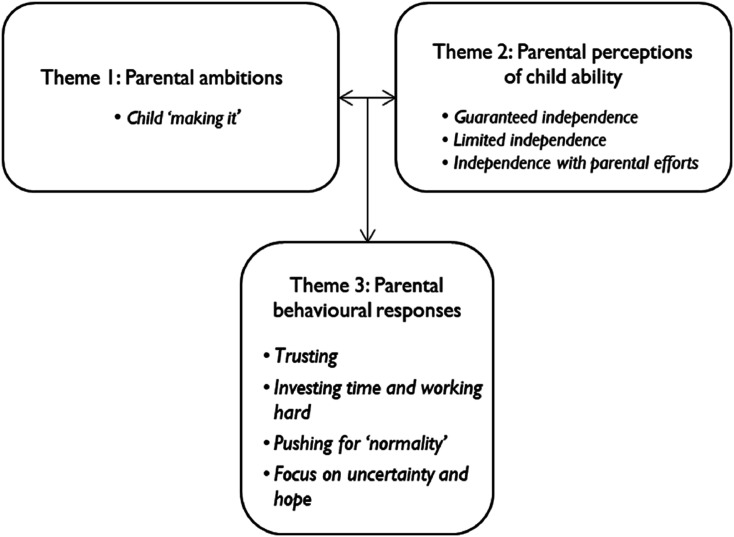
Main themes.

Three interdependent major themes and eight subthemes framed the twenty-two accounts in which the parents reported ambitions for their children (parental ambitions), discussed their children’s characteristics (parental perceptions of child ability), and how they had supported their children to reach those ambitions (parental behavioural responses), in a way in which commonalities within those descriptions emerged. The three major themes were distinct but interdependent, as parental perceptions of child ability and ambitions influenced the way in which parents talked about their parenting behaviour (Figure 1).

In discussing their ambitions for their children (Theme 1), parents’ perception of their child ‘making it’ was a construct of independent, sociable, and happy adult functioning. The notion of ‘future’ was prominent in the parents’ accounts as what their child would be was not yet achieved. Parental belief in their child ‘making it’ was influenced by the parent’s perception of child ability (Theme 2). Three groups of parents were identified: (1) parents who believed their children would become happy, sociable, and independent individuals, (2) parents who did not foresee their children becoming independent adults, and (3) parents who perceived that, although their children were experiencing challenges presently, with extensive parental efforts they would reach happy, sociable, and independent adult life. Parents described varying, somewhat overlapping, behavioural responses to support their children (Theme 3). Although some parents described ‘trusting’, non-intensive parenting behaviours, parents who anticipated their children to have challenges with future independence described intensive parenting practices to support their child’s development. Parents described ‘investing time and working hard’, ‘pushing for ‘normality’, and ‘focusing on uncertainty and hope’.

This overall framework is discussed in detail below. Words in text within single quotation marks indicate that the terms/phrases used are chosen by the parents. Longer quotes are followed by interviewees’ identifier (number), pseudonym, child disability level (none/mild; moderate; severe), and the type of school (junior; secondary; SEND) in brackets.

### Guaranteed independence

Parents who expected no challenges in their child’s future independence described child characteristics of physical and emotional health, showing confidence and being social.

*‘…for her to be healthy physically and mentally, to me, that’s more important than anything. I suppose I want her to do well at school, but more than that I want her to be happy’* (23, Ms Sphalerite, none/mild, secondary).

*‘She’s very independent. She goes to school, she comes home. She’s very confident to go and come back by herself’* (17, Ms Amazonite, none/mild, secondary).

The sense of ‘trusting’ in their child’s abilities allowed these parents not to *‘put too much pressure on’* and they emphasised their child *‘making their own choices’* in terms of education and future profession. A mother stated the following:

‘*...whatever she chooses to do I will encourage and support her*’ (5, Ms Sillimanite, none/mild, secondary).

Although these parents described themselves having the primary responsibility to *‘push and shove’* to ensure their child’s needs were met, for example, to secure extra educational support for their child at school, the parents did not perceive these efforts as the key factor in ensuring their child’s future competence, but rather that the ‘successes’ would originate from their child:

‘*I think if he puts his mind to it, he’ll be able to do it*’ (18, Ms Onyx, moderate, junior).

Although these parents had *‘kept on eye on*’ their child’s development over the years, an initial sense of *‘worry’* following from the *‘unexpected’* EP birth had shifted to a sense of relief as their children had met their developmental *‘milestones’*. Parents described feeling *‘lucky’, ‘grateful’, and ‘fortunate’* in relation to their children’s outcomes and did not, for example, attribute these to their own parenting efforts. A parent explained the following:

‘*...I always say “[child name] I feel you will be successful, and you’ll be a great person.” And she says, “even if I’m working as a cashier,” I say “yes, take five steps, you’ll be a manager ((laughs))”’* (9, Ms Garnet, moderate, junior).

On occasions, parents described that regardless of knowing that *‘all was okay’*, they were still conscious of what their child had *‘gone through’* in relation to being born EP, which had on occasions altered their parenting behaviour making them *‘softer’* by letting their children *‘get away with more’*. A parent recalled her response to a teacher’s feedback of her child’s slow school progress: ‘We just think, well he’s here. He’s happy’ (25, Ms Jasper, none/mild, secondary).

### Limited independence

The parents of children with limited independence focused on discussing their child’s behaviours and abilities with an ambition of their child becoming *‘slightly more independent’* and *‘not rely on [the parent] quite so much’*. Parents perceived caregiving needs to increase with increasing child age as behaviours became harder to control and the children deviated further away from the socially expected behaviour. A mother described her child’s occasional *‘tantrums and strops’* as follows:

‘*...he has got probably worse as he’s got older, and obviously the bigger he’s got, the worse it gets because he’s bigger and stronger*’ (20, Ms Spinel, none/mild, SEND).

*‘...I can see the difference really with his peers and that he does struggle at school still’* (24, Ms Rhyolite, moderate, secondary).

Parents of children with limited independence were not attributing their challenges to ‘causes’ such as the EP birth. Yet parents’ accounts featured a sense of ‘going along’ with it, which appeared as an awareness that their extensive caregiving efforts would always be required. A mother discussed her child’s care needs as follows:

‘*She seems to have got harder work as she’s got older. But I suppose we’ve always done it, so I suppose it’s no different*’ (28, Ms Aquamarine, severe, junior).

The challenges that the parents with children with limited independence described were commonly linked to the extensive caregiving they provided and to the thoughts of their parental responsibilities continuing ‘indefinitely’. Ms Turquoise’s child needed fulltime one-to-one assistance in all their self-care needs:

*‘...I think with a child like this you get stuck in time ((laughs)) so for the last eleven years I’ve been changing diapers and I don’t foresee that I would stop doing that anytime in the future’* (2, Ms Turquoise, severe, SEND).

On occasion, the ‘indefinite’ parental responsibility tied to limited child independence was described as ‘ideal’. Ms Quartz had raised her EP twins virtually alone which had given her a sense of achievement which she perceived as a success. She reflected on the time following EP birth:

‘*…I was aware the situation was real, and this is a massive deal, and there’s only me to do it. But looking back now, I did it so well. People tell me, you’re brilliant, you’re so good with them’* (10, Ms Quartz, moderate & none/mild, junior).

Ms Quartz portrayed an image of ‘adjusted independence’ for her children as *‘never leaving home’* where she would always *‘look after them as their parent’*. Although her children were *‘big, strapping lads’,* they experienced challenges with speech and fine motor skills. The adjusted independence that she perceived as ‘ideal’ might be supportive to her children’s future competence as social adults. EP adolescents’ subtle developmental challenges may hinder their educational aspirations, impact future earning potential, and promote prolonged family dependence (Hallin et al., 2012). By considering her children’s potential challenges with independence, Ms Quartz had interwoven her own ‘ideal’ parental role to fit the adjusted independence she projected for her children.

### Independence with parental efforts

Parents who perceived their children to have challenges in being happy, sociable, and independent, but who believed they would reach independence in future, described their parental efforts as necessary for their children to reach adult competence.

#### Investing time and working hard

For many of these parents the EP birth had been merely a *‘tragic start’,* that had set them back on the route to raising a happy sociable independent adult, which now meant that they had to work *‘extra hard’* to guarantee their children’s success. ‘Investing time and working hard’ ensured the child’s *‘functioning’* and *‘managing school’* which, for example, required the parents to *‘almost train’* their child to *‘behave in a socially acceptable way’*. These parental efforts were referred to as a *‘job’* requiring *‘a lot of time’* and being *‘committed as a family’*. Following from these efforts, however, the child would ‘make it’. A mother described her role as follows:

*‘I see myself as doing this until she’s eighteen, and when she’s eighteen, do you know what, she’ll be all right*. *No one will know*. *She’ll function okay, and she’ll have the basics [*...*] if I put this work in now for the next seven years, she’s gonna have a life where she can actually be the person that she’s supposed to be’* (7, Ms Kyanite, none/mild, junior).

The concept of *‘supposed to be’* may refer to Ms Kyanite’s loss of what she had hoped her child to be (Landsman, 1998), as well as a reference to what a ‘typical’ average child ‘should be’ (Faircloth, 2020) and to parental efforts to ‘repair’ the effects of the risk exposure of EP birth (Kantrowitz-Gordon et al., 2016). Another family described the following:

*‘We’ve invested a lot of time [to caring for their child] and we’ve been committed as a family to that, and I think [child name] has benefitted enormously from that’ *(29, Ms and Mr Jade, moderate, junior).

By ‘investing time and working hard’, these parents gained a sense of control over their children’s outcomes allowing them to work towards achieving the goals for their children and to ensure that their children would *‘fulfil their potential’* and not *‘miss out on’* anything in comparison to children with no special needs or disabilities. A parent described functioning as their child’s *‘caseworker’*:

*‘I think the thing that I’m trying to avoid with SEND plan and all this sort of stuff, I don’t want him to miss out on things, because he was born early. I want him to have the same chances as every other child’* (n. 24, Ms Rhyolite, moderate, secondary).

These parents rarely discussed their children *‘making their own choices’* as did those who perceived their children’s future independence as guaranteed. In contrast to the parents of children with limited independence, although expressing a sense of ‘going along with it’ at present, these parents expected their parenting efforts to eventually cease when independent adult functioning was achieved as was evident in Ms Kyanite’s quote above.

#### Pushing for normality

Contrary to the parents of children with limited independence, parental perception of child characteristics as ‘nearly like others’ among these parents further provoked parental efforts to *‘push’* their child to achieve age-appropriate skills as future adult competence was perceived achievable (Valle, 2017). Ms Iolite felt that because her child, despite their challenges, was *‘quite bright’,* if she did not *‘teach him to act like an average child’* she would be *‘giving up’* on their potential. Commonly these parents compared their EP children’s abilities to those of healthy siblings or classmates, not adjusting expectations due to the preterm birth. This comparison further provoked their parental efforts. Ms Iolite described the following:

*‘I’d like him to grow up in the same way, as much as the other children in his year are and have the same interests, because I just don’t want him to always be so far behind’* (6, Ms Iolite, severe, junior).

#### Focus on uncertainty and hope

Following from prolonged uncertainty in child outcome characteristic of extreme prematurity, some parents in the present study were still expecting a change in their child’s abilities and for them to achieve ‘normality’ even over a decade after the EP birth. For Ms Fire Agate, the uncertainty of her child’s developmental outcomes had nurtured *‘optimism’* and hope for a *‘turning point’,* after which she could return to her ‘own’ life:

‘...*now that he’s going to secondary [school] they’ve advised for him to be diagnosed. Because now he’s not being linked to anything* [...] *I think it’s reached a time now; I just want to know*. *I want to plan for my future*. *Will he need me or is he going to have a turning point*. *I’ve this optimism within me that I’ll catch up with whatever I was pursuing at some point’* (11, Ms Fire Agate, severe, junior).

Parents who experienced uncertainty in relation to their child’s abilities may have found it challenging to accept the altered parenting context, as their perceptions of themselves as parents and their perceptions of their children remained ambiguous (Scorgie et al., 2004). For these parents, the ‘point of realisation’ (or *‘turning point’*) had not yet occurred (Valle, 2017).

## Discussion

This study explored how parents constructed ‘parenting’ their prematurely born child entering adolescence. We found that parents who believed their child would achieve independence had a trusting outlook on their child’s future, attributing it to their child’s qualities rather than their own parenting efforts. These parents did not see their actions as ‘essential’ for their child’s future success, which is encouraging, as perceiving themselves as the ‘essential’ caregiver is linked to increased parental stress and reduced life satisfaction (Rizzo et al., 2012; Shaw et al., 2021). Contrary to our findings, both quantitative (Pyhala et al., 2011) and qualitative investigations (Kantrowitz-Gordon et al., 2016) have reported increased intensive parenting for preterm children. In the present study, the children’s older age might have contributed towards the parents’ ‘trusting’ experiences. As children approach adolescence, parents may naturally reduce their involvement, reflecting developmental appropriateness. This aligns with broader trends showing decreased intensive parenting practices with increasing child age among British families (Yerkes et al., 2019).

Parents who expected their children to face challenges in achieving future independence exhibited various parenting behaviours aligned with elements of parental determinism. Parents who described ‘investing time and working hard’ perceived their parenting behaviour as a means to promote their child’s outcomes long term (Kanieski, 2010). Their responses reflected the notion that with more parental effort, their child’s future competence could be guaranteed, indicating a direct causal impact of parenting on the child’s success (Dermott, 2012; Gillies, 2020). Although similar perceptions have been reported among parents of morbidity-free preschool-aged children (Smyth and Craig, 2017), they particularly align with findings among families with younger preterm children where parents aim to compensate for and ‘repair’ their child’s developmental challenges (Kantrowitz-Gordon et al., 2016). Our findings suggest that comparable intensive parental efforts among EP parents may persist into early adolescence.

Parents did discuss their children’s EP birth, although the adverse birth experience was not a common feature in these parents’ accounts during early adolescence. Contrary to the suggestion that EP birth might create a long-lasting parental perception of child vulnerability (Widding et al., 2020), this was not evident in these parents’ interviews. Studies on parental experiences following preterm birth have generally set the birth and hospitalisation as a starting point, inviting parents to recount their birth stories (Kantrowitz-Gordon et al., 2016; Widding et al., 2020; Wilson and Cook, 2018) concluding that parents may recall the birth as an emotionally charged experience many years later. Yet these studies have a methodological limitation as parents were recruited through methods (e.g. advertisement posters/leaflets) that may have attracted those with particularly strong experiences. Contrarily, our recruitment was researcher-led, offering the study to parents with a wide range of experiences, making our study sample more diverse. Varying parental experiences reported in our study emphasise that the EP population represents a heterogenous group of families, many of whom may not consider themselves as ‘at-risk’.

Gendered parenting roles were highlighted in the study sample, with women predominantly being the main carers. Missing perspectives of fathers in prematurity research have been noted previously (Schuetz Haemmerli et al., 2020). A finding that women assume most caregiving responsibilities is common (Valle, 2017; Wilson and Cook, 2018), particularly among chronically ill children (Kanieski, 2010). Yet studies among families with children with SEND have presented differing findings, with parents sharing responsibilities (Woodgate et al., 2015). In our study, mothers of children with severe impairments did not discuss shared caregiving. While complex care needs may necessitate extensive parental efforts (Page et al., 2020), there is no inherent requirement for these efforts to fall primarily on mothers (Webster, 2018).

### Limitations

The study’s context may have posed limitations. If parents perceived EP birth as a risk factor for their child’s future, they might have felt compelled to emphasise extensive parental efforts to demonstrate how they had ‘adequately’ responded to this risk (Faircloth, 2010). Parents may have been hesitant to express negative feelings like regret (Moore and Abetz, 2018), especially to a researcher they might have regarded as an ‘outsider’; not an EP parent or as a cohort study ‘insider’. Thus, the finding that extensive parental efforts were perceived necessary among some interviewees could partially follow from these limitations.

Aligned with the dominant discursive framing (Faircloth, 2020), ‘parenting’ in the interviews was largely characterised as a series of actions (focused on ‘doing’ rather than ‘being’). The interviewees accounted for their ‘parenting’ by detailing how they supported and would support their children in achieving their goals. The importance of the child ‘making it’ explained parental actions, offering consistency and continuity to their self-perception as parents. The central theme of parental ambitions might have been influenced by several factors, including the interview topic guide design, the cohort study’s focus on long-term outcomes following EP birth, and the time of transition to secondary education. Nevertheless, none of these limitations directed the parents to discuss these parenting behaviours. Thus, methodological considerations alone do not explain the findings.

It is important to place our findings within the wider context of extreme prematurity. While overall survival is improving (Draper et al., 2021), the prevalence of long-term morbidities among survivors remains constant (Marlow et al., 2021), leading to a growing number of families with children with impairments when services for these children, particularly adolescents, are limited (Willis and Godbold, 2023). When parental responsibility is prioritised, the well-being of these parents becomes a societal concern (Valle, 2017). Extensive parental efforts have been associated with decreased mental health and quality of life (Rizzo et al., 2012). Thus, understanding the long-term experiences of parents of children with complex care needs is pivotal. Further research could explore EP parent’ experiences with varying parental support services, especially among parents of adolescents with severe neurodevelopmental disabilities.

## Implications for practice

Our findings demonstrate the importance for professionals working with EP families to consider parental ambitions and perceptions of child abilities as these impact parenting behaviour and experiences. For example, professionals working with EP families are well positioned to challenge sociocultural framings of parenthood that prioritise sole maternal responsibility (Widding et al., 2016). Healthcare professionals may incorporate discussions about ‘good-parent’ beliefs into clinical practice (Weaver et al., 2020).

## Conclusions

This study is the first in the United Kingdom to explore EP parents’ experiences in adolescence and the consequences of parental determinism. Parental ambitions and perceptions of their child’s abilities influenced their parenting behaviour, with a strong desire for their child’s independent adulthood. While some parents adopted ‘trusting’, non-intensive parenting practices, those expecting challenges in their child’s future independence practiced intensive parenting. The study highlights the labour-intensive caregiving efforts for children with complex care needs and underscores the urgent need for supporting parents of children with long-term morbidities, especially in the context of extreme prematurity.

## Data Availability

The data that support the findings of this study are available on request from the corresponding author. The data are not publicly available due to privacy or ethical restrictions.*
